# Where Care Converges: Uncovering Shared Experiences of Parents and Service Providers for Adults With Intellectual Disabilities in Times of Austerity

**DOI:** 10.1111/jar.70044

**Published:** 2025-04-23

**Authors:** Rachel Abigail Harrison, Jill Bradshaw, Michelle McCarthy

**Affiliations:** ^1^ University of Winchester Winchester Hampshire UK; ^2^ University of Birmingham Birmingham UK; ^3^ Tizard Centre University of Kent Canterbury UK

**Keywords:** austerity, care, cuts, networks, parent, service

## Abstract

**Background:**

Since the introduction of austerity measures in 2008, funding for care, welfare, services and support systems in the United Kingdom has been reduced. There is little research that explores the experiences of parents of adults with intellectual disabilities and service providers regarding care, relationships and social networks in times of austerity.

**Method:**

Semi‐structured interviews were undertaken with 10 parents of adults with intellectual disabilities and nine managers and leaders of relevant services. Reflexive thematic analysis was used to identify key themes across their experiences.

**Results:**

Experiences suggested four shared key themes in relation to the effects of austerity: distanced relationships; Care Act assessments being used to make cuts; deteriorating health and wellbeing; and participants feeling forced to become fighters.

**Conclusions:**

Whilst previous research has considered the adversarial nature of relationships between parents and service providers, this study suggests they can experience shared difficulties under austerity.

**Summary**

- Challenges in accessing support: the study found that austerity measures have led to significant reductions in care and support services, making it harder for families of individuals with intellectual disabilities to access needed assistance.
- Importance of social networks: parents and service providers identified social networks as vital sources of support, filling in gaps left by formal services and fostering a sense of resilience and community during times of financial strain.
- Emotional and practical strain: both families and service providers are experiencing increased emotional and practical challenges as they adapt to reduced resources, often feeling stretched to meet needs effectively.
- Impact on Policy and Research: the findings highlight the urgent need for policy changes to improve support structures for people with intellectual disabilities, families and service providers. For the research community, this study underscores the importance of exploring social networks as a critical resource in care delivery during economic hardship.

## 1 Background

In 2008 the Global Financial Crisis, labelled as ‘the most severe since the Great Depression of the 1930s’ (Burton 2016, 1) led to political and fiscal choices being made by successive U.K. governments. Austerity policies, designed to adjust the economy ‘through the reduction of wages, prices and public spending to restore competitiveness’ resulted in significant cuts to welfare and social care budgets (Blyth 2015, 2). Austerity impacted on all those receiving care and support, including those with intellectual disabilities (NIHR SSCR 2020; The King's Fund 2021). Recent increases in government funding to the National Health Service (N.H.S.) and to Local Authorities (L.A.s) since the Covid‐19 outbreak were not considered adequate to fill either previously existing or current social care gaps between funding and people's assessed needs (NHS Digital 2022; The King's Fund 2023).

Research is limited regarding the impact of austerity on the wellbeing of people with intellectual disabilities, their families and carers (Malli et al. 2018; Committee on the Rights of Persons with Disabilities 2016). In addition, the relationship between the requirements for equality and protection of rights suggested under the United Nations Convention on the Rights of Persons with Disabilities (2006) and austerity in the United Kingdom is poorly understood. Our literature review (Harrison et al. 2021) highlighted the crucial role that relationships and social networks play in positively influencing health and wellbeing and the ability to live an ‘ordinary life’. The social networks of adults with intellectual disabilities are typically comprised of family and staff, often serving as positive interpersonal relationships (Sullivan et al. 2016). Research regarding the responses of service providers and families to the effects of austerity in relation to social networks and care is therefore vital if we are to effectively understand and address their health and wellbeing, and by extension, the wellbeing of adults with intellectual disabilities.

Service providers are defined as charities and private organisations providing care, support, services and welfare to people with intellectual disabilities and/or their parents, such as physical care, emotional support, domiciliary care, accommodation, day services, activities and advocacy. These organisations are typically commissioned by L.A.s to provide a set amount of specific support to assessed individuals. In the United Kingdom, funding for all these services has been negatively impacted by austerity. Many of these organisations, therefore, typically now also fundraise to try to meet the shortfall between funds provided by L.A.s and the cost of service provision. The limited research available suggests that service providers' responses to austerity can differ across countries and cultures. In Canada, for example, Courtney and Hickey (2016) found workers responded by forming coalitions linked to unions. In Greece, healthcare workers battled austerity by redoubling their efforts to provide high‐quality services (Kerasidou et al. 2016). In the United Kingdom, leaders and managers of services can often find themselves in difficult relationships with funders (Harris and Roulstone 2011; Mansell 1996). They can struggle to provide basic services in times of cuts (Bradshaw et al. 2018). Research has explored relationships within service teams and less frequently between staff and adults with intellectual disabilities (Beadle‐Brown et al. 2015; Mansell 1996) yet more research is needed on the relationships between leaders and managers of services and their funders in times of austerity. Further research is also required to understand the experiences of parents of adults with intellectual disabilities and service providers in times of austerity in the United Kingdom and globally.

Parents of adults with intellectual disabilities are often the most influential relationship in the lives of people with intellectual disabilities and yet are often overlooked in academic literature. This can instead focus on the financial and emotional ‘burden’ that parents of young children with intellectual disabilities can face (Mahon et al. 2019), which can be overlooked by L.A.s and society more widely (Bauer and Sousa‐Poza 2015; Egan and Dalton 2019). Forrester‐Jones (2021, 109) suggests cuts made by L.A.s, and a L.A. focus on younger parents can ‘inadvertently’ increase this ‘burden’ for parents. Relationships between parents of any age and L.A.s have previously been reported as being poor, often adversarial and as systems‐led rather than person‐centred (Power 2009; Walmsley et al. 2017; Hamilton et al. 2017).

In England, during this time of austerity, The Care Act (2014) was introduced in order to address the perceived unfairness of previous policies under which eligibility for services was felt to have relied on a ‘postcode lottery’ dependent on L.A. funding levels and decisions (Newton and Browne 2008; Newton et al. 2006; Slasberg and Beresford 2017). The Care Act was designed to consider the needs of people with intellectual disabilities and their carers (usually parents).

L.A.s were tasked with bringing in this Act whilst simultaneously saving money. Under The Care Act, eligibility for support requires assessment to be made about whether a person's needs arise from a physical or mental impairment or illness; whether their needs mean they are unable to achieve two or more specified outcomes and whether there is a consequential likelihood of significant impact on wellbeing. Eligibility under The Care Act is not dependent on membership of a labelled group; instead, criteria for eligibility are based on promoting wellbeing and preventing crisis.

While parents in Gant and Bates study reported they were “cautiously optimistic” about The Care Act (Gant and Bates 2019, 432), other studies have suggested that budgetary pressures may threaten carers' rights (Fernandez et al. 2020; Coderre‐LaPalme et al. 2021; Symonds et al. 2018).

This study aimed then to consider the views and experiences of parents of adult sons/daughters with intellectual disabilities and of service providers around care, relationships and social networks in times of austerity.

## 2 Method

While a wider study (Forrester‐Jones et al. 2021) considered the views of 150 adults with intellectual disabilities around the effects of austerity, this article focuses only on the views and experiences of parents and service providers interviewed as part of that study.

Semi‐structured interviews allow participants to explore a particular topic in ways that might be meaningful for them in that they are in control of what they choose to share (Holstein et al. 2013). This may be particularly pertinent for those, as in this study, who are typically underrepresented in research whose views are seldom elicited or heard regarding topics of interest to them. Accordingly, semi‐structured interviews were conducted with 10 parents and nine service providers between September 2017 and February 2019. Questions were created with a project advisory group aiding this study as part of the wider project. The group included parents and service providers. Questions centred around participants' experiences of cuts to care, support, services and welfare and the effects of these cuts on themselves and adults with intellectual disabilities. These questions are provided as Appendices 1 and 2.

Participants lived in locations in the South, the Southwest, and the Northwest of England. Interviews were arranged at a time and place of the participants' choosing. In order to protect anonymity, pseudonyms are used throughout, and location details are general.

For parents, eight interviews were undertaken with individuals and one interview was with a married couple interviewed jointly. Interviews lasted from 46 min to 2 h 17 min. Total interview time was 12 h 6 min and the average length of the nine interviews was 1 h 24 min. Inclusion criteria were that participants had to be the parent (either adoptive or biological) of an adult with intellectual disabilities and had to have a continuing role in their adult son/daughter's care. Parents were aged in their 50s through to their 70s. Sons and daughters were all adults aged in their 20s through to their 50s. All parents had experienced cuts to their welfare payments from the government, which negatively impacted them and their sons/daughters in terms of available resources. All parents reported that their sons/daughters had experienced loss of their services, care and support paid for by their L.A. in terms of hours per week a service was offered and in terms of quality of support offered. Four parents reported their son/daughter had been moved from residential care (24‐h care paid for by the L.A.) to Supported Living accommodation (small group/individual accommodation in ordinary communities, where the majority of costs are paid for by central government), with negative results on both sons/daughters and parents. Parent characteristics are provided in Table 1 below.

**TABLE 1:** Parent characteristics.

Participant pseudonym	Role	Son/daughter interviewed for this study?	Living with son/daughter?	Location	Age	Marital status	Interview type
Janet	Mother to a daughter	No	Yes	South of England	70s	Widow	Lone (with break for lunch) in her home
Karen	Mother to adopted son and daughter	Yes, both	No	South of England	50s	Separated	Lone in her home
Camille	Mother to a daughter	No	Yes	South of England	50s	Divorced	Lone in her home
Dana	Mother to two non‐disabled sons and an adopted daughter	No	No	South of England	70s	Widowed now living with partner	Lone (with break for lunch) in her home
Fred and Frances (married couple interviewed together)	Mother and father to a son with intellectual disabilities who died in the last 10 years, aged in his 20s; and a non‐disabled daughter	No	No	North‐west of England	70s	Married, living together	Joint in a charity building
Robert	Father to two adopted sons	No	Lives with one son	South of England	70s	Married, living together	Lone in his own home
Teresa	Mother of two sons and one daughter. One son had intellectual disabilities	No	Yes	South of England	70s	Widow	Lone (with lunch) in her home

For the nine service providers, five interviews were with individuals and two interviews were each with a pair of participants. Interviews lasted from 20 min to 1 h 39 min. Total interview time was 9 h 54 min and the average length of the seven interviews was 1 h 36 min. One service provider (SR2) provided services to the adult son of one parent (Robert). All other parents and service providers were independent of one another. Inclusion criteria were that participants had to provide some form of care, welfare, support or services to adults with intellectual disabilities and be commissioned by a L.A. or government agency to provide this. All service providers reported loss of commissioned hours of service for all the people with intellectual disabilities they supported, difficulties with fundraising, and heightened concerns over welfare payments for the adults they supported. Service provider characteristics are provided in Table 2 below.

**TABLE 2:** Service provider characteristics.

Participant pseudonym	Role	Location	Organisation type	Interview type
NWP	Manager	Northwest England Small, deprived town	Charity signposting and information service	Lone
ST1	Treasurer	South England Large, deprived town	Charity providing services to people with intellectual disabilities	With ST2
ST2	Job Club Manager	South England Large, deprived town	Charity providing services to people with intellectual disabilities	With ST1
SV1	Residential Home Manager	South England Rural location near a small, deprived town	Charity providing services to people with intellectual disabilities	With SV2
SV2	Residential Home Deputy Manager	South England Rural location near a small, deprived town	Charity providing services to people with intellectual disabilities	With SV1
SR1	Supported Living Service Manager.	South England Rural location	Charity providing services to people with intellectual disabilities	Lone
SR2	Chief Executive Officer	South England Rural location	Charity providing services to people with intellectual disabilities	Lone
GA	Government agency worker	South England	Government agency	Lone
SA	Theatre Company Manager	South England Affluent city	Theatre company charity	Lone

All participant interviews were audio‐recorded and transcribed except for two service providers (SV1 and SV2). As per their wishes, notes only were taken with accuracy later agreed with those participants.

### 2.1 Recruitment and Sampling

There are ‘no magic formulas’ regarding sample size in qualitative research (Braun et al. 2018, 9), but often relatively small sample sizes of between nine and 17 participants can be used to better gain meanings across datasets and in‐depth analyses (Braun and Clarke 2020; Hennink and Kaiser 2022). Vasileiou et al. (2018) raised their concerns regarding the perception that ‘small’ sample sizes could often be understood as ‘insufficient’. They argued, however, that aiming to purposively recruit a small number of participants is intrinsic to the data adequacy of those qualitative studies, which aim to investigate commonalities across experiences in any level of depth.

In order to recruit participants, national and local charities, organisations and groups, including support groups of and for disabled adults and their families, were contacted via email. A national conference aimed at the target groups was visited and study materials were provided to interested parties. Possible participants were visited and all relevant information regarding the study, including consent forms, information sheets and debrief information was provided and explained. Consent was gained from all participants before interviews took place.

### 2.2 Analysis

The choice to use critical realist ontology and contextualist epistemology with inductive reasoning and a qualitative research design created opportunities to consider analytic research methods for this study. Reflexive thematic analysis is becoming a common way to consider the social worlds of people using health and social care services (Braun and Clarke 2006, 2021; Silverman 2020). Reflexive thematic analysis has many advantages including its flexibility, accessibility to new researchers, opportunities for ‘thick description’, ability to allow social and psychological interpretations of data and opportunities for it to be used in policy development (Braun and Clarke 2021, 95). It can ‘provide a rich and detailed, yet complex, account of the data’ (Braun and Clarke 2006, 78). Reflexive thematic analysis allows for data and understandings to be interpreted and reinterpreted, building up a hermeneutic, layered and textured analysis of the possible realities and worlds of participants. Braun and Clarke's (2006) six‐step process was therefore applied to the analysis of the data collected in this study and themes were created which served to answer the research aim and to highlight the complex nature of parents' and service providers' experiences in times of cuts. First, the audio‐recordings were listened to carefully and repeatedly. Then the audio‐recordings were listened to alongside the verbatim transcripts. In this way, it was possible to become familiarised with the data in both audio and written formats. For participants who were not audio‐recorded, agreed written notes were repeatedly examined. In the second stage, initial codes were inductively generated based on phrases that were of importance to the participants. As is common in reflexive thematic analysis, in this initial coding stage, there were no prior specific assumptions or theories which were considered, instead the data were analysed inductively. Initial codes were generated, which included initial exploratory descriptive codes, linguistic codes and conceptual codes. These were then considered in relation to the study aims. In the third stage, the codes were collated into potential themes and data was gathered which related to each theme from across the dataset. In the fourth stage, themes were considered on two levels: firstly, the coded extracts and secondly, the entire dataset. Internal and external homogeneity were considered in order that themes had validity in terms of their similarity within a code; and the degree to which codes in different themes could be clearly established as different from one another. In the fifth stage, themes were defined and named. This involved ongoing analysis in order to refine the specifics of each theme, to consider the overall story the analysis told, and to generate clear definitions and names for each theme. In the sixth stage, results chapters were written as a scholarly report in order to finalise the analysis of the selected extracts. Braun and Clarke advised any extracts should be ‘vivid and compelling’ (Braun and Clarke 2021, 87). This stage provided a final opportunity for analysis, relating back to the research question and literature.

### 2.3 Rigour

In order to safeguard quality, the principles of sensitivity to context, commitment and rigour, transparency and coherence and impact and importance (Yardley 2000, 2008) were applied to this study. Memos and a reflexive journal were kept by the first author throughout the research process on all aspects of the study (Levitt et al. 2017). These were used to reflect on personal and professional positioning, on values and on the process of undertaking and writing the research. The first author had previously managed care homes for adults with intellectual disabilities; for example, dealing with adults with intellectual disabilities, parents, L.A.s so acknowledging these previous experiences and the effects they may have on the research process was vital. Similarly, very distressing experiences were expressed by participants in both groups. This also required exploration and reflexive engagement within the research team, both so that researchers were supported and to ensure themes were created which were not biased toward only the most emotive topics. Methods to reduce power inequalities were implemented throughout the study (Kvale 1996). Braun and Clarke's 15‐point checklist for ensuring quality and rigour in reflexive thematic analysis (Braun and Clarke 2006, 96) was also applied and as such, reporting of research in this study contains often informal language and is written in a tone which best reflects the meanings and intentions of the participants. The use of equal numbers of verbatim quotes from participants allows the reader to make decisions about the hermeneutic claims being made and themes were included only if they were present in all participants' responses.

### 2.4 Ethics Statement

This research project gained ethical approval from the Health Research Authority Social Care Research Ethics Committee (SCREC) on 4 May 2017: REC 17/IEC08/0009; IRAS ID 216910 and the Tizard Centre Ethics Committee on 27 October 17. Consent was gained from all participants before interviews took place.

## 3 Results

Four key themes were created. These were: Austerity Created Negative Relationships; Austerity and Reduced Eligibility; Austerity was Detrimental to Health and Wellbeing; and Having to Adopt an Unwanted Identity of Fighter. These overlapping themes are discussed below. For clarity, names are used to identify parents, whilst letters signify service providers. Numbers relate to the corresponding line numbers in the transcription for each participant.

### 3.1 Theme 1. Austerity Created Negative Relationships

This theme considers the commonalities around which participants felt their relationships with governments and L.A.s had deteriorated and become distanced as a result of austerity.

All participants described the adoption of austerity policies as leading to a perceived increasingly uncaring attitude by governments, L.A.s and their workers.

For participants, living under austerity was described as a keen sense of accelerating loss; particularly the loss of feeling that they, or adults with intellectual disabilities, were cared about and the loss of any personal connection to a supportive professional. They described this change in relationships negatively ‘suddenly it became aggressive’ (Dana, 575). Austerity was experienced painfully by participants, who described its effects as a perpetual feeling of ‘a thousand cuts happening in small slices’ (SR2, 225). The sense of constant and accelerating loss and stress was substantial as a sense of being cared about was eroded under austerity. Participants felt this lack of care was endemic, and led by politicians ‘we have a government who don't really care’ (Janet, 240).

The adoption of an attitude/ethos of cuts and resultant distancing meant that many parents and service providers felt their efforts to achieve a level of normality for themselves and for adults with intellectual disabilities in terms of social networks and relationships were futile ‘the way that it's organised it's impossible to do that’ (Dana, 494). Service providers agreed, ‘care should be person centred, now they are trying to say it can't be person centred’ (SR1, 376–377). ST1 explained ‘the one‐to‐one support work that we do is for social interaction, it must be the most vulnerable aspect’ (ST1, 206–207).

Participants described previously positive relationships with Social Workers who were known to them and acted as family advocates. Some parents had previously experienced negative relationships. All though reported their relationships with L.A.s, Social Workers, and Care Act assessors as deteriorating as a result of austerity policies, to the point that participants no longer felt known or valued. Participants described, for example, the effects of the change from having relationships with known workers to having to engage with inaccessible ‘hubs’. These hubs were described as a single point of contact, which those seeking support or communication with their L.A. must use, typically to a different call‐handler each time:

the net result was if you had a learning disability … you had to go through the Hub, nobody else … if you managed to find a number that sneaked you into County Hall … as soon as somebody realised it was Social Services, you were straight back to the Hub (Fred, 682–689).

Participants also reported the introduction of what they felt were excessively onerous administration tasks which were felt to indicate a lack of trust in parents and service providers and to be designed to deter people from trying to access support or funding. SR1 felt the amount of paperwork now required was ‘absolutely mind‐numbing’ and described chasing L.A.s for thousands of pounds of unpaid invoices as ‘an absolute nightmare’ (SR1, 790, 381–382). The deficit‐model nature of the excessive paperwork parents had to complete about their sons/daughters was described as ‘heart‐breaking’ (Teresa, 591–592).

These changes in relationships were felt to have negative effects on all involved, including L.A., Health and welfare staff ‘the whole system is designed to make people disconnected from their work and the people that they're supposed to serve’ (Barbara, 569–571). Dana agreed:

it's a big conflict of interest for Social Workers because on the one hand, they're supposedly trained to, erm, get people help, and then, on the other hand, when they actually start work, they're trying to stop people getting help. So that doesn't really work, does it? (Dana, 575–578).

### 3.2 Theme 2: Austerity and Reduced Eligibility

This theme considers the ways in which participants felt assessment under The Care Act was used to make cuts.

Participants perceived an inequality in funding reductions, reporting that cuts disproportionately affected them and people with intellectual disabilities relative to other groups, which they felt exacerbated the disparities and barriers they reported. Despite previously experiencing improvements in societal perceptions under *Valuing People* and *Valuing People Now* policies (DoH 2001; DoH 2009), under austerity, participants reported feeling that adults with intellectual disabilities were now perceived as inconvenient burdens with reassessment under The Care Act seen simply as a targeted cost‐cutting tool. Fred (208–212) explained “Health didn't want him because it cost them money, Social Services didn't want him because it cost them money” while GA felt “it's all about the money in the budgets” (GA, 45).

Participants explained there were several ways in which assessment was used as a way to make targeted cuts. Firstly, participants felt Social Workers and assessors were now wilfully ignoring previously accepted evidence “They have rewritten the history” (Janice, 819).

Secondly, Social Workers and assessors were felt to be actively misinterpreting The Care Act in order to make cuts. GA explained “more imaginative interpretations are … [used to] … justify why someone may not be eligible for, say, a supported employment service” (GA, 18–21).

The third way participants felt reassessment was designed to make cuts was through false assumptions about abilities and identities. Participants explained that adults with intellectual disabilities were assessed as if they were simply temporary burdens on the State, with support considered to be a short‐term stepping‐stone to an identity of complete independence. This assumption created a false impression of ability, leading to reassessments that removed much‐needed support. Robert explained:

our son walked in and he was quite well‐mannered and said, ‘Hello’, shook hands and then she had a few words with him and he walked out of the room and she said, ‘Well, he seems okay’. And, you know, it was a sort of comment, throwaway comment, that made us want to tear our hair out with everything that was happening in this house every day (Robert, 369–374).

This was reported as disabling and to have negatively impacted the wellbeing of both adults with intellectual disabilities and parents.

### 3.3 Theme 3: Austerity Was Detrimental to Health and Wellbeing

This theme reports the experiences of participants regarding the various ways in which they felt health and wellbeing was being negatively impacted by austerity for themselves and for adults with intellectual disabilities.

The effects of austerity on the health and wellbeing of participants and adults with intellectual disabilities was felt to be overwhelmingly negative. The language of feeling exhausted, screwed down, untrusted, degraded, not believed, unwanted and excluded was used by all participants in this study. High levels of stress were reported, with all participants reporting negative impacts of cuts on the health and wellbeing of themselves and others. Parents felt that as a result of services being cut ‘to an inch of its life’ (Camille, 354) they now bore ‘the brunt of the caring role’ (Robert, 394). This led some participants to feel that the lives of adults with intellectual disabilities and their parents were “slipping away” unnoticed (Janet, 234–235).

Participants also reported an escalation of inequalities in the avoidable health conditions that adults with intellectual disabilities now experienced and a corresponding perceived decrease in concern by a range of professionals. Frances (759–760) described the response she received when asking for help with her son's health difficulties, being told by a health professional, ‘well it's your own fault he's still alive, you shouldn't be such a good nurse’.

Conditions included self‐neglect, diabetes, severe dental pain, toenails growing into feet, poor diet and weight gain (ST2, SV1&2, NWP, SR1). After one man with intellectual disabilities had his 1 h a week of support cut, he was supported through an unfunded friendship group, which SR2 had created. Through this group, SR2 then found that this man had an untreated urine infection:

cause he didn't know how to contact his doctor. You know, it's as basic as that…. It turns out he'd had it for a year and, you know, who cared? Makes me so cross [tearful]…. If he'd had, you know, just somebody come and see him once a week, it could have been sorted (SR2, 338–342).

Participants felt the early deaths of parents and adults with intellectual disabilities were inevitable. Three parents in this study expressed feeling unbearable pressure, expressing extreme thoughts of murder/suicide, though thankfully none carried this out, ‘no‐one is actually counting the end product … which is premature death’ (Barbara, 829–830). NWP agreed:

natural wastage, that's how the government sees it. It's cheaper. … I don't think anybody cares … I think if I walk through town tomorrow and there was 15 people dead in the street, I don't actually think it would change anything (NWP, 924–921).

The social networks and wellbeing of adults with intellectual disabilities were also impacted by cuts. Exhausted parents had neither the financial, emotional, nor physical energy to maintain their own or their sons/daughters' networks and wellbeing. Being able to spend time outside of the family home was a ‘luxury’ (Robert, 529). When networks could be accessed, these were felt to be important for maintaining any sense of wellbeing. Having social networking opportunities such as advocacy groups then cut by L.A.s was considered by participants to be another way in which cuts across different areas of care and support systems could interact to reduce wellbeing. Participants highlighted the ‘tragic irony’ (GA, 658) that policy and legislation ostensibly promoted the importance of positive measures to improve the relationships, social networks, health, and wellbeing of adults with intellectual disabilities and their parents but in times of austerity, practice appeared to them to be having the opposite effect.

### 3.4 Theme 4: Having to Adopt an Unwanted Identity of Fighter

This theme highlights the ways in which participants felt their identities had changed as a result of austerity.

Participants described feeling relentlessly attacked under austerity. The perpetual conflict in their relationships with governments, L.A.s, Health and other services wore down their resolve to fight back. Participants felt this constant ‘onslaught’ was part of a deliberate strategy ‘there's this feeling that the noose is getting tighter … that things are closing in’ (Janet, 377–378). ST1 explained that previously positive and realistic discussions about funding their services had been commonplace within relationships with their L.A.s., characterised by mutual respect and a shared desire to ‘do good’ but now, ‘the council … can screw that supplier down to the price they want to pay’ (ST1, 270–271).

Service providers felt they were expected to treat adults with intellectual disabilities as commodities over which they must compete. Providers felt they were therefore fighting on several fronts: for their survival, for the rights of adults with intellectual disabilities, against governments and L.A.s and against one another. One provider explained how this change in identity from ‘good’ human charity worker to ‘business animal’ fighting for funding felt:

because there are so many of us at this trough, isn't there, trying to get money, it's that we are amongst the first of these pigs to get to the trough rather than somewhere back in the queue. I don't have that experience; I'm just a volunteer and the world has changed, hasn't it? (ST1, 339–342).

Participants felt their concerns were ignored unless they made formal complaints against L.A.s (SR2). Even then, they perceived that L.A.s did not appear to show an understanding of the needs of adults with intellectual disabilities, their parents, the function of charities, the ways in which services were priced, or how to effectively plan a budget (NWP, SR2, ST1, GA, Barbara, Robert, Janice).

In addition, all parents in this study reported they felt they had to fight back against not only cuts but also against feeling they were being lied to, being lied about, and some felt punished when they fought back against their perceived attackers.

Barbara and Dana were clear that Social Services staff had ‘blatantly’ lied to them and about them regarding assessments under The Care Act ‘in order to legally reduce, they had to tell lies actually’ (Barbara, 520, 317–318). Three parents who had adopted their sons/daughters felt they had been lied to about the nature of their sons/daughters' levels of need and the level of support they could expect. They had not expected to have to fight for basic support they felt they deserved (Robert, Dana, Karen).

Five parents (Janice, Barbara, Teresa, Fred and Frances) related their experiences of feeling deliberately ‘targeted’ for “onslaught” (Barbara, 476, 433) in a ‘mixture of austerity … and … personal vendetta’ (Janice, 1114). They felt this was because they had complained about L.A.s. When Frances and Fred protested against cuts at a Council meeting, they reported being ejected by armed police (Fred, 384), for example. This was reported in the local press at the time. After making complaints, Janice explained ‘I was secretly taken off the respite list for two years. I found out later from a new Social Worker’ (Janice, 252–253).

Barbara explained that the feelings of being attacked and needing to fight back on behalf of oneself and of adults with intellectual disabilities could be exhausting:

but it gets harder and harder, and you have to keep digging into that well of hope when someone keeps poking at you …. Poking, poking, poking, poking, poking. And the thing about this is, and what really hurts … is this is being done to the most vulnerable in the land. These are people who can't fight back. That's shameful [pause] (Barbara, 558–563).

Service providers agreed, feeling that ‘fighting’ (SR1, NWP, SA, SR2) and ‘battling’ (ST1, NWP, SR1) on behalf of those who could not fight for themselves, in a variety of ways, had become normalised. In response, with little or no funding, several providers set up friendship, advice, advocacy and hate crime groups for adults with intellectual disabilities who had had all their care and support cut (SR1, SR2, NWP). These and other social networks were described as providing ‘a little bit of ammunition’ and support for fighting back against failing systems (Camille, 313). She explained why this ammunition was needed:

I think the main thing is to say that every time you go for funding it is an absolute fight, and you have to be a Tiger Mummy if you know what I mean, and really fight for everything that you can get. And I think if you're not that natured, it's very hard and it's very stressful (Camille, 21–24).

The results of this perceived uncaring, adversarial approach under austerity were felt to be fundamentally detrimental at both individual and societal levels:

there is a real risk that if we sort of accept what's happening now as the norm, that we will just go backwards and adults with learning disabilities will not be full members of our society; they will be second‐class citizens that are forgotten and not cared about. And I think that would be just a terrible retrograde step (SR2, 414).

## 4 Discussion

This study explores the impact of austerity on care and social networks from the perspectives of 10 parents and nine service providers. Our literature review found that the opportunity for an ‘ordinary life’ for adults with intellectual disabilities was reliant on family and staff. This study has extended that review by highlighting the ways in which some parents and service providers feel austerity has negatively impacted their relationships, care and social networks and those of adults with intellectual disabilities.

Key findings have raised issues around the perceived effects of austerity on the nature of relationships, on the purpose and experiences of assessment, on wellbeing, and on identities. That these findings are reported by both parents and service providers suggests people in these groups may have more in common than previously thought.

In terms of relationships, this study highlights the feelings shared by parents and service providers regarding what they saw as the deliberate design of systems to create distance between individuals and the support systems they rely on. Power (2008) previously found this to be the case for parents. In our study, however, both service providers and parents reported that access to support was increasingly restricted through difficult‐to‐access hubs and the withholding of essential information at a time when austerity policies were felt to have increased their caregiving responsibilities, leaving them too exhausted to maintain social connections. Parents felt that L.A.s' cuts to advocacy groups further isolated them. This suggests the perception of a systematic attempt to block access to support, moving beyond previous research, which suggested that barriers were often inadvertent (Forrester‐Jones 2021) or overlooked (Bauer and Sousa‐Poza 2015; Egan and Dalton 2019).

Ironically, policies intended to promote wellbeing were felt, in times of austerity, to have contributed to loneliness and isolation. Participants felt uncared for, untrusted, and excluded. Early deaths and deteriorating health and wellbeing were seen as inevitable outcomes of these austerity measures. Additionally, service providers at managerial level reported high levels of stress and burnout, a new finding in the academic literature, which adds to Stevens et al.'s (2021) and Thomas and Rose's (2010) work regarding frontline staff and to Bradshaw et al.'s (2018) work regarding managers. This study underscores how austerity has negatively impacted health and wellbeing for all involved, creating a climate of distress and deteriorating mental health.

The Care Act (2014) introduced new eligibility criteria aimed at reducing the ‘postcode lottery’ of care provision. It sought to enhance well‐being, prevent crises, and save money. However, findings from this study suggest that austerity measures were felt to have undermined these goals. Both parents and service providers reported heightened risk of crisis for themselves and adults with intellectual disabilities, raising concerns that austerity might yield short‐term savings but create greater long‐term costs. This extends the work of Gant and Bates (2019) as rather than feeling, ‘cautiously optimistic’ about The Care Act (Gant and Bates 2019, 432), parents and service providers in this study actively criticised both The Care Act itself and its implementation.

In this study the reassessment process, meant to promote wellbeing, was instead perceived as a mechanism to reduce care. Far from preventing crises, reassessments were seen as a means of cutting costs, thereby disabling those in need. This study questions the purpose of reassessments under The Care Act, highlighting how they often resulted in decreased support for people whose needs had remained the same or even increased. This gives detail to Fernandez et al. (2020)'s study regarding the ways in which budgetary pressures may negatively affect carers' rights and aligns with academic literature (Coderre‐LaPalme et al. 2021; Symonds et al. 2018) indicating that reassessment processes can often be focused on reducing social care budgets rather than accurately assessing needs.

Relationships between L.A.s and parents have often been reported in academic literature as adversarial, with parents feeling the need to adopt the identity of fighters (Power 2009; Walmsley et al. 2017). Parents in our study also gave examples of needing to change their identities to fight back against perceived attacks. However, our study adds the finding that service providers too can feel the need to adopt roles of fighters, for similar reasons and in similar ways. Service providers' battles in our study were against cuts to their own services and the services of adults with intellectual disabilities, against their changing identities, and against the application of detrimental policies by L.A.s. This also appears to be new in the academic literature beyond those found in other studies such as Malli et al. (2018) Kerasidou et al. (2016) and Courtney and Hickey (2016).

The following recommendations are, therefore, made.

### 4.1 Recommendations for Policy

Government and L.A.s should evaluate the financial and emotional effects of austerity policies on budgets, their staff, adults with intellectual disabilities, parents, and service providers. Policy should be written in partnership with adults with intellectual disabilities, parents, and service providers. Additionally, a promise from governments (with appropriate ring‐fenced funding for L.A.s) that every individual with intellectual disabilities will have appropriate support throughout their lifetime from someone who knows them well.

### 4.2 Recommendations for Practice

Any concerns government and L.A.s feel regarding the effects of austerity should be shared with adults with intellectual disabilities, parents, and service providers in order to enhance understanding of experiences. Better communication from governments and L.A.s regarding the purpose of reassessment would be beneficial in order that all parties are clear on the purpose of assessment before assessment takes place. Training should be provided to governments and L.A.s by adults with intellectual disabilities, parents, and service providers regarding the nature of intellectual disabilities and associated needs in order that future policy and practice reflect the lived experiences of these groups. In addition, the formation of a care advocacy collective made up of parents and service providers would be beneficial in order to challenge decisions and promote partnership.

### 4.3 Recommendations for Research

A longitudinal study using the same measures to understand experiences over time would add to understandings of the effects of austerity. Greater inclusion of the experiences of adults with significant intellectual impairments could serve to enhance understanding of their experiences. The inclusion of research participants who have not experienced deterioration in their relationships with L.A. workers would also enable better understanding of the factors which influence positive relationships. The inclusion of qualified and unqualified assessors in research and the factors which influence their judgements about eligibility for care would aid better understanding of their experiences of assessing under austerity.

## 5 Limitations

This study, like most, has limitations. Interviews were conducted pre‐Covid, since when the U.K. government and austerity measures have changed. Participants chose to take part and were dissatisfied with their experiences, which may have influenced results. Many parents who declined participation cited time constraints, possibly skewing the study towards parents and providers who were articulate, used to fighting for services, and who had a grasp of strategic and practice issues. As with all qualitative studies, small numbers of participants provide opportunities for in‐depth considerations of a few experiences. A different quantitative study, which considers the experiences of more parents and service providers may yield different results.

Future research could benefit from a more diverse sample, across various socio‐economic backgrounds, ethnicities, geographical locations and L.A. areas, including more adoptive parents and the views of funders, L.A. workers and commissioners. Research which used a smaller sample from just one group could more meaningfully use Interpretive Phenomenological Analysis and unstructured interviews. A Grounded Theory approach in this or future studies could provide opportunities for different themes to be created.

Finally, this study did not explore the extent to which austerity policies may have interacted with the protections required under the Convention on the Rights of Persons with Disabilities (2006). This could be a useful focus for future research.

## 6 Conclusion

This study adds to the limited research on austerity's effects, suggesting that policies and practices under austerity have contributed to distanced relationships, a sense of disempowerment, and diminished health and wellbeing among parents and service providers. We suggest that there is scope to consider not only the differences between parents and service providers but also the possible opportunities for coming together to share experiences to better understand and more effectively address current and future decision‐making processes around the adoption of austerity policies together. The health, wellbeing, care and social networks of all involved may thereby be enhanced.

## Conflicts of Interest

The authors declare no conflicts of interest.

## Data Availability

The data that support the findings of this study are available from the corresponding author, Dr. Rachel Harrison, upon reasonable request. Data access will be provided to researchers subject to review of their proposed use and adherence to confidentiality agreements, ensuring compliance with ethical guidelines. Due to the sensitive nature of the data involving individuals with intellectual and developmental disabilities, some data may be available in a de‐identified form to protect participant privacy.

## Appendix 1 Indicative Interview Schedule for Service Providers

1. Has your organisation/service been affected by austerity?
2. If it has, in what ways?
3. Does austerity affect the service you provide?
4. Does austerity affect the people who use your service and their carers?

## Appendix 2 Indicative Interview Questions for Parent/Carers

1. Have you or your adult son/daughter been affected by austerity?
2. How do any effects make you feel?
3. How was your life before austerity measures?
4. How is your life now?
5. Has austerity impacted on your social network?
6. Has austerity impacted your job?
7. How do you organise the care for your loved one now?
